# Cases in Mercy Hospital—Gynæcological Wards

**Published:** 1871-12

**Authors:** 


### CLINICAL REPORTS.

### CLINICAL CASES IN MERCY HOSPITAL—GYNÆCOLOGICAL WARDS.

Services of PROF. BYFORD,

CASE I. ECZEMA OF THE SCALP.—The affection known as scald head may appear at any time of life, though it is most frequent among children. It is a popular notion that the disease is contagious, and while this is uncertain, its possibility may be guarded against appropriately. In the beginning of this case, small vesicles appeared in different parts of the head, filled with pellucid contents, which soon became yellow and mixed with pus, pustules being formed in two weeks. Few vesicles came together, but they were scattered all over the scalp and, if untreated might spread so as to occupy the entire surface. With the developement of the pustules pus is effused and forms a cake of scab on the head; when this comes off the integument below is bare of hair, the pustules having formed round the roots of the hairs and destroyed the bulbs. Our patient presents small naked patches especially about the occiput; the lymphatic glands of the neck and about the ears are swollen, undoubtedly from the irritation of poison brought from the inflamed parts through the lymphatic vessels.

The treatment must be local, the general health of course being attended to as in other cases. The impression is prevalent among the people that scald head is dependent on an impurity of the blood, and that this condition must be remedied. The only way to cure the affection is to change the condition of the inflamed parts and make the inflammation acute. The hair is to be cut or shaved off, and poultices put on to remove the scabs; sulphur or tar ointment may sometimes be applied to kill any parasites. Then the scalp should be washed every day with strong soap suds, and once in two days be bathed with strong solution of citric acid.

---

Case II. Vesico-Vaginal Fistula.—A lady who has been operated on twice before, the last time about two years ago, again presents herself to undergo the operation for vesico-vaginal fistula; the opening has been reduced to a very small one by the previous operations and its complete closure is attempted by the usual procedure. A case operated on two weeks previously is reported a complete success, and the patient is about to go away relieved.

---

Case III. Cancrum Oris.—This girl, six years old, exhibits a rare and very destructive disease. The child was attacked four weeks ago with chills and high fever; she lives near the stock yards, a vicinity which is exceedingly malarious, and the fever was probably cured by quinine. The mother says their doctor cured the fever in four or five days, and then gave some powders and a bottle of yellow medicine, but the mouth became sore and the substance eaten away around three teeth, which fell out. The doctor discontinued the medicine and recommended a wash of alum water.

A week ago the cheek began to swell, and in four or five days became very tense and prominent. In forty-eight hours a blister appeared on the inside of the cheek, surrounded by a dark spot; it soon sloughed and the ulceration has extended through the cheek and eaten away a large part of the right side of the face. The gangrene is approaching the eye which is inflamed and possibly may ulcerate. The child has just been brought to the Hospital, and probably will soon die, though cures are sometimes made, about one case in twenty surviving.

Cases of cancrum oris are frequently found to have been exposed to malaria, as in the present instance. The diagnosis of the disease is easy. The gums are generally tumefied, though not for so long a time as in this case; a termination is common in the course of five days. An ash colored blister appears on the inside of the cheek, soon ulcerates through the cheek which then becomes gangrenous.

The treatment is to touch all the advancing ulcer with nitric acid in order to get a new and plastic inflammation. Tonics and nutritious food are to be given in as large quantities as the patient can take. Quinine gr. ij is to be given every four or six hours, and a dressing of carbolic acid and glycerine applied.

---

CASE IV. ECZEMA DURING TEETHING.—A skin disease frequently affects children during the stage of teething; it generally begins with little blisters on the face which extend up onto the scalp, and there have much the character of eczema. Eruptions commenced on this child nearly a year ago when he was five months old, at the time the first tooth appeared; after the tooth came through he was better; the eyetooth produced especial trouble in the skin, he has had good health otherwise. A pimple first appeared on the top of the scalp, and then others came out all over the head, and a few on the body. The matted scabs which cover the head like a hood are caused by the effusion, and could be taken off by poultices when little pustules would be found on every square quarter of an inch.

In regard to treatment the Professor dreads to see the affection dried up during teething, and thinks this a great injury. He regards the skin disease as a kind of safety valve, and does not remember a cure of it without the child being made sick; while children with sore heads do not have summer complaints. After teething is finished the skin disease generally disappears, or can easily be cured; no scars are produced. The head is to be cleaned and annointed with sweet oil; if the eyes are affected blisters may be applied behind the ears, the only treatment allowable. The Professor would not take the responsibility of curing the skin disease now, before the completion of teething.

